# Transferring Occupational Therapy Knowledge to Village Health Volunteers to Enhance Activities of Daily Living Among Older Adults in Community‐Based Elder Care: A Qualitative Study

**DOI:** 10.1155/oti/8750838

**Published:** 2026-05-13

**Authors:** Autchariya Punyakaew, Suchitporn Lersilp, Napalai Chaimaha, Kewalin Panyo, Supawadee Putthinoi

**Affiliations:** ^1^ Department of Occupational Therapy, Faculty of Associated Medical Sciences, Chiang Mai University, Chiang Mai, Thailand, cmu.ac.th

**Keywords:** activities of daily living, community-based care, occupational therapy, older adults, Village Health Volunteers

## Abstract

**Introduction:**

Thailand′s rapidly aging population highlights the urgent need for sustainable strategies to support older adults′ independence in activities of daily living (ADLs). Occupational therapists (OTs) play a vital role; however, their limited availability restricts community‐based service delivery. Village Health Volunteers (VHVs), embedded within local communities, provide ongoing support but lack structured guidance in ADL‐focused care.

**Objective:**

This study is aimed at developing occupational therapy–informed practice guidelines that translate professional expertise into culturally relevant recommendations for community‐based eldercare.

**Methods:**

A qualitative design using semistructured interviews was employed with 10 OTs in Chiang Mai, Northern Thailand. Participants were recruited through stratified and purposive sampling across urban, semiurban, and suburban settings. The inclusion criteria required a minimum of 5 years of clinical experience with older adults and direct involvement in ADL training. Data were collected between April and June 2024, transcribed verbatim, and analyzed thematically. Guiding questions were interpreted using the Occupational Therapy Practice Framework: Domain and Process, 4th Edition (OTPF‐4), while additional inquiry explored broader community and cultural themes. Credibility was ensured through rapport building, audio recording, and member checking.

**Results:**

OTs recommended adaptive techniques, environmental modifications, caregiver education, and assistive device integration. Culturally adapted solutions, such as Thai garments, bamboo ramps, and gradual modification of bathing practices, were emphasized to enhance acceptance and sustainability.

**Conclusion:**

This study provides structured, occupational therapy–informed guidelines to support ADLs among community‐dwelling Thai older adults. Empowering VHVs through these recommendations may strengthen community‐based eldercare, reduce caregiver burden, and promote active, dignified aging in place.

## 1. Introduction

Global demographic shifts present unprecedented challenges and opportunities. The World Health Organization (WHO) projects a significant increase in the global population aged 60 and older, rising from 1 billion in 2020 to 1.4 billion by 2030 and reaching 2.1 billion by 2050. This growth is most pronounced in low‐ and middle‐income countries, where approximately 80% of all older people are expected to live by 2050 [1]. While this demographic shift reflects global progress in public health and development, it also underscores the urgent need for health systems and social support networks to adapt to the demands of an aging population [2]. A central priority of aging policy frameworks worldwide, including WHO′s Active Ageing and Decade of Healthy Ageing initiatives, is to enable older adults to maintain independence in ADLs, which are critical markers of health, autonomy, and quality of life [3, 4].

In Thailand, the pace of demographic aging is among the most rapid in Southeast Asia. The Thai Gerontology Research and Development Institute [5] reported that over 18% of the population is now aged 60 years or older, a proportion expected to rise steadily in the coming decades. With aging comes increased vulnerability to physical, cognitive, and psychosocial decline, leading to heightened dependence in basic ADLs such as feeding, dressing, bathing, toileting, and mobility [6, 7]. Recent occupational therapy studies in Thailand have further highlighted that the concept of active aging (defined by the interplay between health, participation, and security) offers a valuable framework for understanding older adults′ engagement in daily life. For example, Punyakaew et al. [8] demonstrated that levels of active aging and patterns of time use among Thai older adults in suburban communities are closely related to their independence and well‐being. Building upon this foundation, Punyakaew et al. [9] developed and validated a culturally grounded Active Aging Index that integrates international concepts with Thai‐specific contextual factors such as community participation, intergenerational relationships, and environmental support. The instrument demonstrated good internal consistency (Cronbach′s *α* = 0.77) and strong test–retest reliability (*r* = 0.89), providing a reliable tool for assessing active aging among Thai older adults and reinforcing the importance of contextually tailored measures in community‐based elder care. These findings collectively highlight that promoting active aging in Thailand requires not only healthcare access but also community engagement and environmental adaptation aligned with older adults′ cultural lifestyles. The Thai government has responded by implementing national screening programs for ADL performance using the Barthel Index, categorizing older adults as socially active, homebound, or bedridden [10]. Despite these efforts, the growing demand for rehabilitation and community‐based support continues to outpace the availability of professional services.

Occupational therapists (OTs) play a central role in eldercare, particularly in maintaining or restoring ADL performance through therapeutic interventions, environmental modifications, and caregiver education. Evidence from international studies demonstrates that OT‐led interventions can significantly reduce functional decline, prevent unsafe living conditions, and improve overall quality of life for older adults [11, 12]. However, Thailand faces a critical shortage of OTs, especially in rural and semiurban areas [13]. Most OTs are hospital‐based and can only provide limited home‐visit services, often as infrequently as two visits per year. Consequently, many older adults and their families lack consistent access to professional support for ADL performance, underscoring the need for alternative mechanisms to extend OT expertise into communities.

One such mechanism is Thailand′s long‐standing Village Health Volunteers (VHV) program, a nationally recognized model of community‐based health support. VHVs, trained under the Ministry of Public Health, are embedded in the communities they serve, providing health promotion, disease prevention, and basic caregiving support [14, 15]. Their roles include conducting home visits, monitoring health behaviors, and assisting bedridden or chronically ill patients. During the COVID‐19 pandemic, VHVs played a pivotal role in sustaining community health services and managing public health risks [16]. Their close proximity and trust within communities position them as vital intermediaries between families and formal healthcare systems.

Despite their contributions, VHVs face several challenges. Studies highlight that VHVs often lack sufficient knowledge and skills in specialized areas such as chronic disease management, rehabilitation, and ADL‐focused caregiving [17, 18]. Training programs are typically ad hoc and lack standardization, leading to inconsistent practices across regions [19]. This gap underscores the necessity of developing structured, evidence‐based guidelines that can be applied by VHVs under the supervision or consultation of OTs. By systematically transferring professional knowledge from OTs to VHVs, it becomes possible to strengthen community‐based eldercare, support family caregivers, and promote sustainable aging‐in‐place.

To address this gap, the present study sought to develop occupational therapy–informed guidelines for enhancing ADL performance among older adults in community settings. Drawing on the clinical experiences and expertise of licensed OTs with more than 5 years of geriatric practice, this qualitative study generated evidence‐based recommendations that reflect both professional knowledge and the cultural realities of Thai communities. Importantly, these guidelines are designed not only to inform OT practice but also to be adapted into practical, culturally sensitive tools for VHVs, who remain the most consistent frontline providers of care in community settings. By bridging the expertise of OTs with the accessibility of VHVs, this study offers a sustainable model for strengthening elder care within Thailand and provides insights relevant to other countries facing similar challenges of workforce shortages and rapidly aging populations.

## 2. Materials and Methods

### 2.1. Study Design

This study employed a qualitative research design aimed at developing evidence‐based occupational therapy guidelines to enhance the functional performance of older adults in ADLs. In‐depth, semistructured interviews were conducted to explore OTs′ professional experiences, therapeutic approaches, and perspectives on ADL‐related interventions. This approach allowed for rich, detailed insights to inform guideline development.

### 2.2. Ethics Statement

This study was approved by the Ethics Committee of the Faculty of Associated Medical Sciences, Chiang Mai University (Approval No. AMSEC‐66EX‐094). All participants provided written informed consent before data collection.

### 2.3. Participants

OTs working in Chiang Mai, Northern Thailand, were recruited for this study. A stratified sampling strategy was initially employed to ensure representation across three practice settings—urban, semiurban, and suburban—followed by purposive sampling to select between eight and 10 participants in total, in line with qualitative research recommendations for achieving data saturation [20]. Inclusion criteria required participants to have a minimum of 5 years of clinical experience providing services to older adults, direct experience in occupational therapy related to ADL training and guidance, and willingness to participate with informed consent, while the sole exclusion criterion was an inability to commit to the full study process.

### 2.4. Data Collection

Data were collected between April and June 2024 using semistructured, in‐depth interviews following the approach of Saunders et al. [21]. An interview guide was designed to elicit participants′ knowledge, strategies, and professional experiences regarding ADL interventions. Questions included the following:

a. “Could you describe your experience with ADL training for older adults? Please elaborate on the interventions and therapeutic approaches you have used.”
b. “From your perspective as an occupational therapist, what evidence‐based strategies or techniques should be suggested to support older adults, family members, and caregivers in maintaining long‐term independence in ADLs?”
c. “What strategies or techniques should be recommended specifically to family members and caregivers to optimize the functional abilities of older adults with ADL limitations?”
d. “How can the professional experiences and recommendations of occupational therapists be transferred into practical guidance for Village Health Volunteers (VHVs) in Thailand?”

All interviews were conducted in a natural, conversational manner to encourage openness. Interviews were audio‐recorded with participants′ permission and transcribed verbatim. Data trustworthiness was enhanced through rapport‐building, clarification of research objectives, and the use of techniques such as probing and summarization. Member checking was conducted by summarizing key points during and after interviews, with participants confirming the accuracy of interpretations.

### 2.5. Data Analysis

Thematic analysis was used to examine and interpret the interview data. Transcripts were read repeatedly to ensure immersion in the data, and initial codes were assigned to meaningful segments. Codes were then organized into broader themes that captured the essence of participants′ perspectives. For Interview Questions (a)–(c), data were deductively coded using the *OTPF-4* [22] as the analytic framework to ensure alignment with established occupational therapy domains. In contrast, data derived from Question (d) were analyzed inductively, allowing themes related to knowledge translation and community implementation to emerge from the data. This approach allowed emergent themes to be interpreted flexibly, reflecting broader community and cultural contexts beyond the OTPF framework. To ensure credibility, member checking was conducted during and after analysis, enabling participants to verify the accuracy and authenticity of the interpretations.

## 3. Results

This qualitative study involved 10 OTs in Chiang Mai, Thailand, representing diverse practice settings: three from urban areas, four from semiurban areas, and three from suburban areas. All participants had more than 5years of professional experience and were actively engaged in community‐based practice, including ADL training and recommendations, interdisciplinary collaboration, and home visits (Table 1).

**Table 1:** Characteristics of participants (*N* = 10).

Participant	Gender	Age (years)	Years of work experiences	Current position	Practice setting
Participant 1	Female	52	31	Occupational therapist	Urban
Participant 2	Female	45	24	Practitioner level	Urban
Participant 3	Female	46	25	Practitioner level	Urban
Participant 4	Female	35	14	Occupational therapist	Suburban
Participant 5	Male	35	14	Occupational therapist	Suburban
Participant 6	Male	42	21	Practitioner level	Suburban
Participant 7	Male	37	16	Practitioner level	Suburban
Participant 8	Female	39	18	Practitioner level	Semiurban
Participant 9	Female	40	19	Practitioner level	Semiurban
Participant 10	Female	34	13	Practitioner level	Semiurban

**Occupational Therapy–Informed Guidelines**

Through thematic analysis, six core domains of ADLs were identified: feeding, grooming, dressing, bathing, toileting, and mobility. Within these domains, distinct themes emerged that reflect OTs′ clinical experiences and professional recommendations. The findings highlight the complicated relationship among individualized care, adaptive methods and techniques, environmental modifications, caregiver involvement, and cultural considerations in elder care. Table 2 summarizes the emergent themes, followed by detailed descriptions.

**Table 2:** The emergent themes of ADL recommendations from OT perspectives.

Theme	Sub‐theme	Representative quotes
1. Feeding: Balancing safety and independence	1.1 Collaborative care for swallowing safety	“We collaborated with speech therapists to evaluate swallowing ability and then trained caregivers to recognize signs of choking….” (P4)
	1.2 Environmental and behavioral adaptations	“I suggested that older adults visit a dentist annually for oral health checkups and maintain regular oral hygiene practices….” (P1)
	1.3 Assistive tools as “game changers”	“I recommended an oval‐lipped cup for individuals with Parkinson′s disease or stroke‐related impairments….” (P2)
2. Grooming: Preserving dignity through adaptive strategies	2.1 Small changes, big impact	“A long‐handled sponge lets them wash their back independently; it′s about dignity.” (P7)
	2.2 Caregiver empowerment	“We suggested stimulating older adults to open their mouths during tooth brushing, verbally guiding them through each step….” (P8)
3. Dressing: Navigating physical and cognitive barriers	3.1 Adaptive clothing innovations	“Choosing garments based on individual abilities is essential. Options include front‐opening shirts….” (P5); “We recommended culturally familiar clothing styles with simple modifications….” (P10)
	3.2 Safety as a shared responsibility	“We teach seated dressing first; standing to pull up pants is a major fall risk.” (P2)
4. Bathing: Bridging tradition and safety	4.1 Rethinking traditional bathing practices	“We suggested that older adults transition to showering rather than using a water scoop….” (P6)
	4.2 The invisible labor of caregivers	“We suggested that caregivers minimize physical strain by avoiding excessive bending….” (P4)
5. Toilet use and hygiene: Balancing privacy and practicality	5.1 Promoting dignified toileting solutions	“Locally available materials such as PVC pipes or bamboo can be utilized to construct grab bars….” (P11)
	5.2 Hygiene as a social determinant of health	“We emphasized the use of wet wipes for post‐toileting hygiene….” (P6)
6. Mobility: Balancing access, culture, and professional guidance	6.1 Addressing environmental barriers	“Ensuring adequate lighting in entryways and bathrooms, along with warning indicators such as red stickers….” (P2)
	6.2 Assistive devices: Gifts and burdens	“Older adults with physical limitations should consider using assistive devices for safe mobility.” (P7)
7. Recommended guidelines when equipping Village Health Volunteers	7.1 Keep it simple	“It′s best to avoid using specialized clinical terms. Use simple, everyday language that people can easily understand; instead of saying ‘postural instability,’ use ‘unsteady when sitting or standing.’” (P3)
	7.2 Facilitating referral to professionals	“I think it would be great to train VHVs to use a simple communication tool like ‘What I saw… What I tried… What I need.’ It′ll help them report situations more clearly when a referral is needed.” (P8)
	7.3 Experiential training of VHVs	“VHVs should be trained through practical, hands‐on activities that prepare them for real‐life situations… Exercises like this promote practical problem‐solving and help build confidence.” (P2)
	7.4 Sustainability in the Thai context	“We should apply the OT guidelines that support older adults with daily activities—especially those designed for VHVs—and connect them with the existing systems in the community. This will help ensure continuity and long‐term sustainability.” (P2)

### 3.1. Feeding: Balancing Safety and Independence

#### 3.1.1. Collaborative Care for Swallowing Safety

OTs underscored the importance of working with other healthcare professionals, particularly speech therapists and nurses, to manage dysphagia and ensure safe oral intake. This collaboration included caregiver education to identify early signs of swallowing difficulty and adapt food textures appropriately.

We collaborated with speech therapists to evaluate swallowing ability and then trained caregivers to recognize signs of choking—such as coughing or a wet vocal quality—and to adjust food textures accordingly. (Participant 4)

#### 3.1.2. Environmental and Behavioral Adaptations

Creating supportive mealtime environments was identified as essential. Recommendations included reducing background noise, using appetizing aromas, and simple modifications such as adjusting bed height or using rolled towels for positioning. Oral hygiene before and after meals was also emphasized to prevent respiratory infections.

I suggested that older adults visit a dentist annually for oral health checkups and maintain regular oral hygiene practices to prevent aspiration pneumonia. (Participant 1)

#### 3.1.3. Assistive Tools as “Game Changers”

Assistive devices such as angled utensils, nonslip mats, and adapted cups were described as crucial for promoting independence despite motor impairments.

I recommended an oval‐lipped cup for individuals with Parkinson′s disease or stroke‐related motor impairments, as it prevents the nose from pressing against the rim and enables easier drinking without excessive head tilting. (Participant 2)

#### 3.1.4. Grooming: Preserving Dignity Through Adaptive Strategies

##### 3.1.4.1. Small Changes, Big Impact

Minor modifications, such as electric toothbrushes or long‐handled sponges, were reported to enhance independence and self‐esteem.

A long‐handled sponge lets them wash their back independently; it′s about dignity. (Participant 7)

##### 3.1.4.2. Caregiver Empowerment

Caregivers were encouraged to use verbal prompts rather than completing grooming tasks for older adults, thereby promoting autonomy.

We suggested stimulating older adults to open their mouths during tooth brushing, verbally guiding them through each step of hygiene tasks such as face washing, hair combing, and shaving. (Participant 8)

#### 3.1.5. Dressing: Navigating Physical and Cognitive Barriers

##### 3.1.5.1. Adaptive Clothing Innovations

Participants emphasized aligning clothing choices with cultural norms and individual needs. While Western‐style adaptive clothing, such as Velcro fasteners, was sometimes resisted, alternative strategies included using front‐opening shirts, larger buttons, or culturally familiar Thai garments with discreet adaptations.

Choosing garments based on individual abilities is essential. Options include front‐opening shirts for individuals with shoulder stiffness or larger buttons for easier fastening. (Participant 5)

We recommended culturally familiar clothing styles with simple modifications, such as loose‐fitting Thai garments, to respect preferences while maintaining independence. (Participant 10)

##### 3.1.5.2. Safety as a Shared Responsibility

Safety in dressing routines was stressed, with strategies such as seated dressing recommended to reduce fall risk.

We teach seated dressing first; standing to pull up pants is a major fall risk. (Participant 2)

#### 3.1.6. Bathing: Bridging Tradition and Safety

##### 3.1.6.1. Rethinking Traditional Bathing Practices

In the Thai context, traditional water‐scooping practices were recognized as culturally significant. OTs recommended gradual transitions to safer methods, such as seated showering, while respecting cultural norms.

We suggested that older adults transition to showering rather than using a water scoop and recommended pump‐style soap dispensers and long‐handled bathing brushes for safety. (Participant 6)

##### 3.1.6.2. The Invisible Labor of Caregivers

Caregiver burden was highlighted, with the recommendation of assistive devices such as shower chairs to reduce strain.

We suggested that caregivers minimize physical strain by avoiding excessive bending when assisting older adults during bathing. (Participant 4)

#### 3.1.7. Toilet Use and Hygiene: Balancing Privacy and Practicality

##### 3.1.7.1. Promoting Dignified Toileting Solutions

Bedside commodes and bathroom modifications were recommended, using low‐cost local materials when possible.

Locally available materials such as PVC pipes or bamboo can be utilized to construct grab bars, reducing costs while enhancing accessibility and safety. (Participant 10)

##### 3.1.7.2. Hygiene as a Social Determinant of Health

Proper toileting hygiene was considered essential for preventing infections.

We emphasized the use of wet wipes for post‐toileting hygiene to enhance self‐care and reduce caregiver burden. (Participant 6)

#### 3.1.8. Mobility: Balancing Access, Culture, and Professional Guidance

##### 3.1.8.1. Addressing Environmental Barriers

OTs emphasized that mobility often begins at the threshold of the home, where entryways and uneven surfaces pose critical safety risks. To overcome these barriers, they recommended simple structural modifications such as constructing ramps from locally available materials, including bamboo, wood, or cement blocks. These adaptations not only reflect Thai traditions of resourcefulness and reliance on community craftsmanship but also provide affordable solutions for families with limited financial resources. In addition, therapists stressed the importance of enhancing visibility by installing adequate lighting and using culturally familiar warning indicators, such as brightly colored stickers or fabric markers, to highlight uneven surfaces and reduce accidents.

Promoting safety within residential environments can be achieved through the installation of adequate lighting in high‐risk areas such as entryways and bathrooms, alongside the use of low‐cost, locally sourced materials. For example, bamboo—a readily available and culturally familiar resource—can be painted in bright colors such as red or orange to serve as visual warning indicators on uneven surfaces. (Participant 2)

##### 3.1.8.2. Assistive Devices: Gifts and Burdens

While mobility aids such as canes, walkers, and wheelchairs were recognized as essential for maintaining independence, OTs noted that many older adults resisted their use due to cultural perceptions that such devices symbolized weakness or dependency. To address this barrier, therapists recommended caregiver education and community awareness strategies to normalize the use of assistive devices. By framing mobility aids as tools that enable dignity, safety, and social participation, rather than as symbols of frailty, OTs sought to foster acceptance and adherence. OTs play a key role in bridging professional expertise with community practices, ensuring that interventions are not only clinically effective but also socially and culturally acceptable.

Older adults often resist the use of assistive devices due to cultural perceptions that associate such equipment with disability and social stigma. This attitude significantly influences their willingness to adopt mobility aids. (Participant 3)

Older adults with physical limitations should consider using assistive devices for safe mobility. (Participant 7)

#### 3.1.9. Translating OT Expertise Into VHVs

Translating the specialized knowledge of OTs into practical guidance for VHVs is key to sustainable, community‐driven elder care in Thailand. The goal is to empower VHVs with an “OT lens”—a way of seeing and solving problems that focuses on function, safety, and dignity.

##### 3.1.9.1. Keep It Simple

Communicate clearly and accessibly. Avoid complex clinical terminology so that caregivers and community volunteers can apply the guidance in everyday situations without needing professional training.

It′s best to avoid using specialized clinical terms. Use simple, everyday language that people can easily understand; instead of saying “postural instability,” use “unsteady when sitting or standing.” (Participant 3)

Provide simple and practical advice. For example, if the home doesn′t have handrails, suggest using bamboo or rope available in the household to create temporary support. This can help older adults walk with more confidence and safety. (Participant 7)

##### 3.1.9.2. Facilitating Referral to Professionals

VHVs play a vital role as the frontline link between older adults and professional care services. To ensure timely and appropriate care, they must be able to recognize situations that require professional intervention beyond basic home adjustments. A simple, structured communication tool can empower VHVs to make effective referrals.

I think it would be great to train VHVs to use a simple communication tool like “What I saw… What I tried… What I need.” It′ll help them report situations more clearly when a referral is needed. (Participant 8)

##### 3.1.9.3. Experiential Training of VHVs

The “OT for VHV” training modules are designed as short, highly interactive workshops—typically half‐day or full‐day sessions—facilitated by OTs. These sessions emphasize experiential learning through role‐playing and hands‐on practice, allowing VHVs to engage directly with real‐life scenarios. To deepen relevance and connection, the training incorporates real stories and photographs from the local community, helping VHVs relate the learning to familiar contexts and recognize the value of their role in promoting safe, functional living environments.

VHVs should be trained through practical, hands‐on activities that prepare them for real‐life situations. For example, participants might be given a wobbly chair and a few cushions and asked, “How would you make this safer for an older adult?” Exercises like this promote practical problem‐solving and help build confidence in making low‐cost environmental adjustments using materials commonly found in the home. (Participant 2)

Using pictures in basic OT guides for supporting older adults with ADL really helps VHVs understand and apply the advice in real situations. It makes things clearer and easier to follow, especially when they′re working in the community. (Participant 6)

##### 3.1.9.4. Sustainability in the Thai Context

In the Thai context, promoting sustainability in occupational therapy practices means integrating recommendations with existing community systems and resources. Rather than relying on imported or high‐cost equipment, interventions should prioritize materials that are locally available, affordable, and familiar—such as bamboo, cotton fabric, rope, or bricks. These items can be creatively repurposed to support safe mobility, home modifications, or therapeutic activities. Moreover, design suggestions should reflect and respect local lifestyles. For example, encouraging older adults to practice walking in temple grounds or community pavilions not only utilizes accessible public spaces but also reinforces cultural relevance and social inclusion. By aligning therapeutic strategies with everyday environments and materials, VHVs and OTs can foster long‐term adoption and meaningful impact.

We should apply the OT guidelines that support older adults with daily activities ‐especially those designed for VHVs—and connect them with the existing systems in the community. This will help ensure continuity and long‐term sustainability. (Participant 2)

It′s also important to coordinate with the local health‐promoting hospitals so that everyone is working in the same direction and getting the right support. (Participant 10)

## 4. Discussions

The findings of this study highlight the critical role of occupational therapy–informed guidelines in empowering VHVs to support older adults in performing ADLs within the Thai community context. By translating professional knowledge into culturally relevant and practical strategies, these guidelines help bridge the gap between specialized care and community‐based support. This approach aligns with global trends in task‐shifting and community health empowerment, particularly in low‐ and middle‐income countries where access to OTs is limited [23, 24].

The Occupational Therapy–Informed Practice Guidelines for VHVs in Community‐Based Elder Care were systematically organized into six primary domains of ADLs: feeding, grooming, dressing, bathing, toileting and hygiene, and mobility. This classification is consistent with the OTPF‐4 [22], specifically within the domain of ADLs. The recommendations represent a multidimensional approach derived from occupational therapy perspectives, emphasizing not only older adults but also the involvement of families and caregivers. Beyond functional performance, ADLs are closely tied to identity, autonomy, and dignity. Tasks such as grooming extend beyond hygiene to reinforce self‐worth and emotional well‐being. Occupational therapy, therefore, should frame these routines as affirmations of personhood, particularly in cultural contexts where aging may diminish visibility and social participation [25]. While safety‐focused interventions such as seated dressing and the use of assistive devices are essential for reducing risks, they may inadvertently restrict autonomy if applied too rigidly. This tension between protection and independence highlights the need for ethical reflection in clinical decision‐making.

Barriers to assistive device adoption were also identified, including stigma, affordability, and cultural perceptions. Many older adults resist using devices due to pride or beliefs that they symbolize dependency [26, 27]. OTs must therefore advocate for inclusive policies and education that normalize device use and dismantle ableist narratives. Cost remains a significant obstacle, as access to assistive devices in Thailand is largely tied to disability registration, which provides medical and social benefits. However, many eligible individuals remain unregistered, revealing a gap in service coverage [28, 29]. Finally, the study underscores the importance of cultural sensitivity in developing ADL interventions. Interventions that promote Western‐style clothing or modern bathing practices may conflict with Thai cultural norms, raising concerns of cultural imperialism. The locally adapted solutions identified in this study, such as loose‐fitting Thai garments and community‐based environmental modifications, demonstrate the importance of tailoring recommendations to the cultural context. Broader integration of cultural sensitivity into caregiver training is essential for sustained adherence and effectiveness.

Importantly, the study reinforces the value of experiential learning and visual aids, such as real‐life photos and role‐play scenarios, in enhancing VHV understanding and confidence. These methods not only facilitate knowledge transfer but also foster problem‐solving skills using locally available materials [30, 31]. The emphasis on sustainability through system integration, such as collaboration with health‐promoting hospitals and use of existing community spaces, further strengthens the potential for long‐term impact [32]. Moreover, the structured communication tool (“What I saw… What I tried… What I need…”) offers a practical framework for VHVs to report complex cases and initiate timely referrals. This tool supports clearer coordination between VHVs and professional teams, reducing delays in care and improving safety outcomes for older adults [33, 34].

While the guidelines show promise, future research should explore their implementation across diverse regions, considering variations in community infrastructure, VHV capacity, and cultural norms. Additionally, codesigning training modules with VHVs and older adults may enhance relevance and uptake [35]. Ultimately, this study contributes to the growing body of evidence supporting community‐based occupational therapy and offers a scalable model for strengthening aging‐in‐place strategies in Thailand and similar contexts [22, 36].

## 5. Conclusions

This study underscores the central role of occupational therapy in enhancing older adults′ capacity to perform ADLs within community‐based care. The evidence‐based recommendations developed here were systematically organized into six domains—feeding, grooming, dressing, bathing, toileting and hygiene, and mobility—reflecting the breadth of occupational therapy practice as defined in the OTPF‐4 [22]. The emergent themes highlight strategies such as adaptive techniques, environmental modifications, caregiver education, and the integration of assistive devices, all of which are essential for promoting independence and alleviating caregiver burden.

In the Thai context, where the availability of licensed OTs is limited, VHVs represent a critical resource for extending occupational therapy recommendations into everyday community practice. Strengthening the knowledge and skills of VHVs through structured training and culturally sensitive guidelines is therefore vital to advancing sustainable eldercare, supporting families and caregivers, and fostering active and dignified aging among older adults.

## 6. Limitations and Suggestions

This study was limited to data obtained from OTs in Chiang Mai, Thailand, which may constrain the transferability of findings to other regions with different healthcare systems and sociocultural contexts. Furthermore, the exclusion of VHVs′ perspectives restricts insight into their lived experiences, challenges, and training needs in supporting older adults. For future research, further studies should evaluate the effectiveness of these guidelines when implemented by VHVs in real‐world settings, explore the long‐term outcomes for older adults, and investigate the use of digital platforms for delivering scalable training. Collectively, these directions can help build a sustainable, culturally responsive model of community‐based occupational therapy that supports aging in place for older adults in Thailand and other low‐ and middle‐income countries.

## Author Contributions

Autchariya Punyakaew oversaw the entire study and contributed to the drafting and dissemination of the manuscript. Suchitporn Lersilp, Napalai Chaimaha, and Kewalin Panyo conducted the data collection and the data analysis. Supawadee Putthinoi designed the research plan and coordinated the structural framework of the manuscript.

## Funding

The authors acknowledge the financial support provided by the Faculty of Associated Medical Sciences, Chiang Mai University, Thailand (Project No. R67IN00001).

## Conflicts of Interest

The authors declare no conflicts of interest.

## Data Availability

The data that support the findings of this study are available on request from the corresponding author. The data are not publicly available due to privacy or ethical restrictions.
